# The anti-inflammatory effect of the synthetic antimicrobial peptide 19-2.5 in a murine sepsis model: a prospective randomized study

**DOI:** 10.1186/cc11920

**Published:** 2013-01-09

**Authors:** Tobias Schuerholz, Sabine Doemming, Mathias Hornef, Lukas Martin, Tim-Philipp Simon, Lena Heinbockel, Klaus Brandenburg, Gernot Marx

**Affiliations:** 1 Department of Intensive Care and Intermediate Care, University Hospital Aachen, RWTH Aachen University, Pauwelsstr. 30, Aachen, 52074, Germany; 2 Department of Microbiology, Hannover Medical School, Carl-Neuberg-Str. 1, Hannover, 30625, Germany; 3 Division of Biophysics, Forschungszentrum Borstel, Parkallee 10, Borstel, 23845, Germany

## Abstract

**Introduction:**

Increasing rates of multi-resistant bacteria are a major problem in the treatment of critically ill patients. Furthermore, conventional antibiotics lead to the release of bacterial derived membrane parts initiating pro-inflammatory cascades with potential harm to the patient. Antimicrobial peptides (AMP) may kill bacteria without releasing pro-inflammatory factors. Thus, we compared three newly developed synthetic anti-lipopolysaccharide peptides (SALPs) with a broader range of efficacy to suppress cytokine release in plasma and CD14 mRNA expression in organ tissue in a murine, polymicrobial sepsis model.

**Methods:**

A randomized, experimental trial was conducted in an animal research facility. Male NMRI mice (*n *= 90; 8- to 12-weeks old) were randomized to the following six groups: (i) sham operation and parenteral vehicle (NaCl 0.9%) administration (sham); (ii) cecal ligation and puncture (CLP) and vehicle infusion (sepsis-control), (iii) CLP and polymyxin B infusion (polyB), or (iv to vi) CLP and infusion of three different synthetic antimicrobial peptides Peptide 19-2.5 (Pep2.5), Peptide 19-4 (Pep4) or Peptide 19-8 (Pep8). All animals underwent arterial and venous catheterization for hemodynamic monitoring 48 hours prior to CLP or sham-operation. Physical appearance and behavior (activity), plasma cytokine levels, and CD14 mRNA expression in heart, lung, liver, spleen and kidney tissue were determined 24 hours after CLP or sham operation.

**Results:**

Only Pep2.5 significantly enhanced the activity after CLP, whereas none of the therapeutic regimens elevated the mean arterial pressure or heart rate. The strongly elevated IL-6, IL-10 and monocyte chemoattractant protein serum levels in septic animals were significantly reduced after Pep2.5 administration (*P *< 0.001, *P *< 0.001, and *P *< 0.001, respectively). Similarly, Pep2.5 significantly reduced the sepsis-induced CD14 mRNA expression in heart (*P *= 0.003), lung (*P *= 0.008), and spleen tissue (*P *= 0.009) but not in kidney and liver.

**Conclusions:**

Structurally variable SALPs exhibit major differences in their anti-inflammatory effect *in vivo*. Continuous parenteral administration of Pep2.5 is able to reduce sepsis-induced cytokine release and tissue inflammation.

## Introduction

Despite intense effort in basic and clinical research the morbidity and mortality of sepsis and septic shock have remained high. The increase in the resistance pattern of many bacterial blood stream isolates against the most commonly used antibiotics during the past years further hampers adequate clinical management. In recent years the number of multiresistant bacteria isolates has increased and triggered the implementation of an Interagency Task Force on Antimicrobial Resistance (Centers for Disease Control and Prevention, USA). New therapeutic strategies to combat severe infections are, therefore, urgently needed.

Conventional anti-infective agents may kill bacteria but at the same time release bacteria-derived agents, such as lipopolysaccharide (LPS) or lipoprotein (LP), hence causing the devastating consequences of the pro-inflammatory cascades in severe sepsis and septic shock. Antimicrobial peptides (AMP) may kill bacteria without releasing pro-inflammatory factors, but their application may be impeded by high toxicity, hemolysis, nephrotoxicity and neurotoxicity [1]. Therefore, the challenge is to develop synthetic peptide-based drugs on the basis of naturally occurring AMPs in order to treat septic patients effectively without causing harm.

A new series of peptides of amino acid lengths in the range 17 to 23 was developed to adapt to the physico-chemistry of the lipid A part of endotoxins. The structure of these peptides is based on the LPS-binding domain of the Limulus anti-LPS factor as a template and was modified with respect to its capacity to bind and neutralize LPS [2-4]. In sepsis patients this may be an advantage since antibiotics may accelerate the release of pro-inflammatory components increasing the severity of disease [5,6].

These newly designed synthetic anti-lipopolysaccharide peptides (SALPs) with decreased toxicity and a broader range of efficacy are able to neutralize LPS-mediated effects at different degrees *in vitro *as well as *in vivo *[4,7]. In particular, SALPs change the LPS structure into a multi-lamellar structure accompanied by large increases in aggregate size. Furthermore, strong Coulomb interaction between peptide (positive charges) and LPS (negative charges) influence the incorporation of the peptides into the LPS bilayer [4,7].

We hypothesized that our newly developed SALPs are well tolerated when infused intravenously (i.v.) in therapeutic amounts. Furthermore, inflammation indicated by cytokines in plasma and CD14 expression in organ tissue should be decreased in a murine model of polymicrobial sepsis.

## Materials and methods

### Catheterization

After permission of the local animal protection authorities (LANUV, NRW, Germany: Az 8.87-50.10.35.09.044) all mice (male NMRI mice (*n *= 90, body weight 38 ± 3 g) underwent a catheterization procedure. Under general anesthesia (isoflurane 1% to 2% in oxygen/air mix with a FiO2 of 0.3) spontaneously breathing animals were fixed in the prone position. The right neck vessels were exposed under sterile conditions after local anesthesia with 0.2 ml lidocaine 2% (Astra Zeneca, Wedel, Germany) and a central vein catheter (CVC; self-made using sterilized polyethylene tubing with an outer diameter of 0.61 mm) was implanted 1 cm deep in the jugular vein enabling continuous i.v. administration of fluids and drugs throughout the experiment. The CVC was tunneled to the back of each mouse and guided through a flexible plastic tube (Drainobag 40, B.Braun, Melsungen, Germany) to prevent bite damage. A 27G cannula was inserted into the CVC to connect the CVC to the syringe pump. In addition, an arterial transmitter (PA-C10, Data Sciences International, St. Paul, MN, USA) was implanted to measure heart rate (HR) and mean arterial pressure (MAP). The catheter consisted of a transmitter unit and a gel-filled tube which was introduced and fixed in the right carotid artery. The transmitter unit was implanted subcutaneously in the abdominal region. Subsequently, after further local infiltration with lidocaine, the neck was closed by single suture and the mouse transferred back into the cage to rest for 48 hours prior to the induction of sepsis by cecal ligation and puncture (CLP). To prevent hypothermia all animals were kept on a heating pad throughout the surgical procedure.

### Sepsis induction

**Figure 1:**
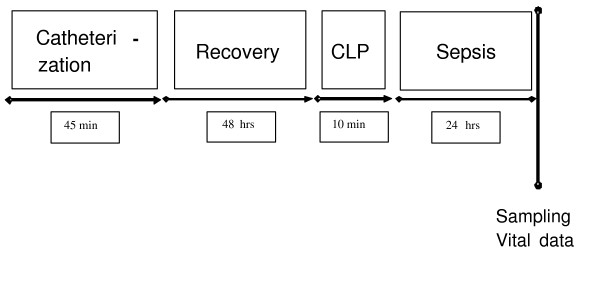
**Course of experimental procedure**. At the end of the observation vital data were recorded and blood and organ tissue were sampled. CLP, cecal ligation and puncture.

General and local anesthesia for CLP was performed as described above. Under sterile conditions a midline laparatomy of 1 cm was performed. The cecum was identified, subtotal ligated approximately 1 cm away from its base and afterwards twice perforated with an 18G needle. Feces were protruded to assure the perforations were opened. Then the cecum was replaced and the abdominal cavity was closed by single suture. The animal was transferred to the cage and reconnected with the i.v.-line to the syringe pump at a rate of 100 μl/hour. All animals were killed after 24 hours. The entire experimental course is depicted in Figure 1.

The animals were randomly assigned to one of six groups:

1. Sepsis-control (*N *= 15): sepsis induction and vehicle (NaCl 0.9%) infusion

2. Sham (*N *= 15): sham operation (laparotomy without cecal ligation) and vehicle infusion

3. PolyB (*N *= 15): CLP sepsis with polymyxin B infusion (1.2 μg/hour Polymyxin B in NaCl 0.9%)

4. Pep 2.5 (*N *= 15): CLP sepsis with peptide 19-2.5 (Pep 2.5) infusion (2.0 μg/hour peptide in NaCl 0.9%)

5. Pep 4 (*N *= 15): CLP sepsis with peptide 19-4 (Pep 4) infusion (pep 4, 2.0 μg/hour peptide in NaCl 0.9%)

6. Pep 8 (*N *= 15): CLP sepsis with peptide 19-8 (Pep 8) infusion (2.0 μg/hour peptide in NaCl 0.9%).

Polymyxin B, considered as the most potent anti-endotoxin agent, was infused in group three to determine LPS-neutralizing effects. The latter three groups were treated with the SALPs. The dose of the peptides had been evaluated in preceding experiments, combining best decrease in pro-inflammatory cytokines without side effects.

All animals received a bolus of 200 μl NaCl 0.9% when transferred to the cage. One animal in the sepsis-control and two animals each in the other groups were excluded before analysis due to loss of CVC or bite damage to the CVC.

### Vital data measurements

The average of the last five measurements of HR and MAP by the arterial transmitter was recorded after 24 hours of sepsis. The physical activity of the mice was recorded independently by two of the investigators who were blinded to treatment before sampling. They used a predefined scoring system ranging from 1 (healthy) to 5 (agony). This scoring system is based on rating physical activity and food intake (see Table 1) using spontaneous activity of the mice, reaction to exogenous stimuli and spontaneous food intake to differ between the grades 1 to 5 [8].

**Table 1:** Scoring system to measure physical activity of mice [8].

Grade	Quality	Criteria
1	Very active	Strong, curious, quick movements, normal food intake

2	Active	Strong, curious, single occasional interruptions in activity, normal food intake

3	Less active	Adequate response to environment, frequent interruptions in activity, slightly decreased food intake

4	Slow	Sleepy, slow activity, severely decreased food intake

5	Lethargic	No activity, motionless, no food intake

### Sampling

After 24 hours of sepsis, the animals were anesthetized, brought to a prone position and the abdomen and thorax were opened. Blood was sampled in pre-citrated syringes and, after centrifugation, plasma samples were frozen following the manufacturers guidelines until measurement. Directly after killing the animal under general anesthesia by cervical dislocation, heart, liver, lung, kidney and spleen were harvested. All organ tissues were snap-frozen in liquid hydrogen until further processing.

### Laboratory measurements

RNA was extracted from frozen tissue (RNeasy Mini Kit, Qiagen, Hilden, Germany), reverse transcripted to cDNA (Quanti Tec, Rev Transcription Kit, Qiagen) and analyzed by real-time-PCR (RT2 real-time SYBR green/rROX PCR master Mix, PA-012, Biorad, Hercules, CA, USA) with a mouse specific primer to CD14 (biomers, Ulm, Germany) using a 7300 Real-time PCR-System (Applied Biosystems, Carlsbad, CA, USA). Due to financial restrictions, we randomly assigned 10 out of 15 samples for PCR diagnosis of CD14 of all organs. PCR was performed using 40 ng of cDNA and standard PCR protocols in 96-well PCR plates (ABgene, Epsom, UK) containing 15 μl reaction/well with ReadyToGo PCR beads (Amersham, Braunschweig, Germany). PCR primers were as follows: CD14: 5'-CAG AAT CTA CCG ACC ATG GAG-3' (forward), 5'-GGA ACA ACT TTC CTC GTC TAG C-3' (reverse). As a control we used beta-actin (actb) with the following primer: 5'-GCT CTT TTC CAG CCT TCC TT-3' (forward), 5'-CGG ATG TCA ACG TCA CAC TT-3' (reverse). Both primers were designed using a freely accessible source [9]. The following conditions were used: 95°C for 3 minutes, then 40 cycles at 95°C for 30 seconds, 57°C for 30 seconds and 72°C for 30 seconds.

Determination of IL-6, IL-10 and monocyte chemoattractant protein-1 (MCP-1) was performed using a cytometric bead array (CBA, BD Biosciences, Heidelberg, Germany). The CBA consists of bead populations with distinct fluorescence intensities with specific capture antibodies. These bead populations were quantitatively measured using a flow cytometer. We have chosen IL-6 and IL-10 as pro- and anti-inflammatory cytokines released by monocytes, macrophages and neutrophils. Additionally, MCP-1 was measured as a chemotactic agent for monocytes and macrophages. These three measures are related to the signal transduction by the CD14-receptor via the Toll-like receptor (TLR) complex.

### Peptides

The SALP-peptides were synthesized with an amidated C terminus by the solid-phase peptide synthesis technique in an automatic peptide synthesizer (model 433A; Applied Biosystems) on Fmoc-Rink amide resin, according to procedures described earlier [7]. The amino acid sequences of the three peptides, Pep 2.5, Pep 4, and Pep 8, used here are shown in Table 2.

**Table 2:** Sequences of the synthetic anti-LPS peptides and molecular weights.

Peptide	Sequence	Molecular weight
Pep19-2.5	GCKKYRRFRWKFKGKFWFWG	2711
Pep19-4	GKKYRRFRWKFKGKWFWFG	2750
Pep19-8	GRRYKKFRWKFKGRWFWFG	2636

### Statistical analysis

Differences in cytokine measures between groups after 24 hours were evaluated by the Kruskal-Wallis test followed by a Bonferroni-corrected Wilcoxon Rank-sum test using SPSS Statistics 20.0 for Windows (SPSS Inc. Chicago, Illinois, USA). The PCR-derived data were analyzed using a relative expression software tool (REST) performing a randomization test. This tool avoids assumptions on distributions and applies a pair wise fixed reallocation randomization test^© ^which reallocates control and sample groups (= pair wise fixed reallocation). The expression ratios are calculated on the basis of the mean crossing point (CP) values for reference and target genes [10]. All data are given as mean ± SD unless otherwise indicated. *P *< 0.05 was considered significant.

Based on previous experiments in human mononuclear cells [7] we assumed a reduction of pro-inflammatory cytokines of at least 30% by application of Pep 2.5 and a weaker effect in the other groups. A total sample size of at least 90 animals was necessary in order to achieve a power of at least 90%.

## Results

### Activity and vital parameters

The activity of mice in the sham-, Pep 2.5- or polymyxin B-treated groups was significantly higher (each *P *< 0.001; Table 3) compared to the sepsis-control group 24 hours after CLP. Animals treated with Pep 4 and Pep 8 showed no differences.

**Table 3:** Activity index, mean arterial pressure and heart rate after 24 hours of sepsis.

	Sham	Sepsis-control	polyB	Pep 2.5	Pep 4	Pep 8
**Activity index (points)**	1.1 ± 0.3^a^	4.4 ± 0.8	3.2 ± 0.5^a^	2.5 ± 0.7^a^	4.0 ± 0.8	4.1 ± 0.7
**Mean arterial pressure (mmHg)**	149 ± 40	126 ± 65	100 ± 27	97 ± 38	92 ± 49	86 ± 29
**Heart rate (1/minute)**	485 ± 197^b^	310 ± 88	364 ± 140	434 ± 196	297 ± 108	354 ± 125

^a^*P *< 0.001 versus control; ^b^*P *< 0.05 versus control. Activity index ranges from 1 (best activity) to 5 (agony). Sham, sham operation with vehicle infusion; Sepsis-control, sepsis with vehicle infusion; polyB, sepsis with PolymyxinB infusion; Pep 2.5, sepsis with peptide 19-2.5 infusion; Pep 4, Sepsis with peptide 19-4 infusion; Pep 8, sepsis with peptide 19-8 infusion; Data given as mean ± SD

There were no significant differences in HR between the treatment groups, although HR was significantly higher in the sham group compared to sepsis-control mice (*P *= 0.016; Table 3). Therapy with Pep 2.5 resulted in a non-significantly higher HR (*P *= 0.082, Table 3). The MAP showed no significant differences (Table 3).

### Plasma cytokines

**Figure 2:**
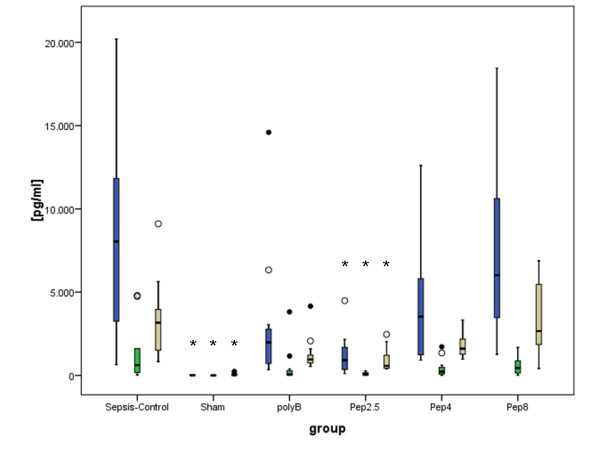
**Plasma cytokines after 24 hours of sepsis**. Blue columns = Interleukin-6; green columns = Interleukin-10; beige columns = Monocyte chemoattractant protein-1; sepsis-control (*N *= 14) = Sepsis with vehicle infusion; Sham (*N *= 13) = Sham operation with vehicle infusion; polyB (*N *= 13) = Sepsis with PolymyxinB infusion; Pep 2.5 (*N *= 13) = Sepsis with peptide 19-2.5 infusion; Pep 4 (*N *= 13) = Sepsis with peptide 19-4 infusion; Pep 8 (*N *= 13) = Sepsis with peptide 19-8 infusion; Data given as box-and-whisker depicting smallest and largest observation, median ± interquartile range (box); * *P *< 0.01 versus control.

IL-6 was increased in the sepsis-control group and significantly decreased in the Pep 2.5-group compared to the sepsis-control group (*P *< 0.001). Treatment with polyB and Pep 4 and Pep 8 did not reduce IL-6 release (Figure 2).

IL-10 was significantly decreased in the Pep 2.5 group (*P *= 0.002) but not in the Pep 4 (*P *= 0.111), polyB (*P *= 0.11) or Pep 8 (*P *= 0.073) groups compared to the sepsis-control group (Figure 2). Likewise, the MCP-1 level was significantly reduced in the Pep 2.5 group (*P *< 0.001), but not in the polyB (*P *= 0.70), Pep 4 (*P *= 0.183) or Pep 8 (*P *= 0.718) groups compared to the sepsis-control group (Figure 2).

### Gene expression

**Figure 3:**
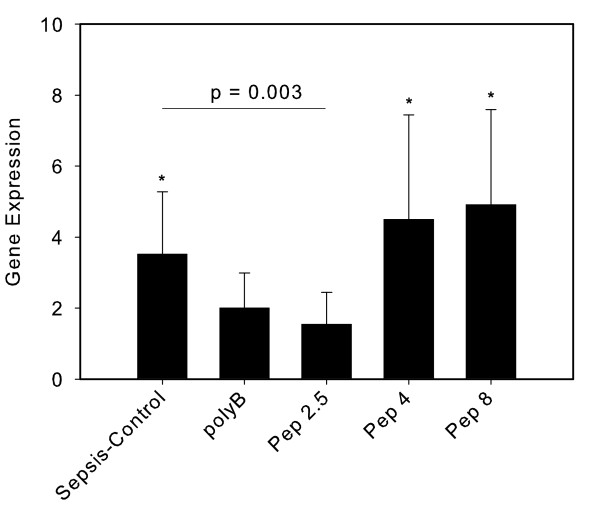
**CD14 expression in heart tissue after 24 hours of sepsis**. Sham (*N *= 10) = Sham operation with vehicle infusion; Sepsis-control (*N *= 10) = Sepsis with vehicle infusion; polyB (*N *= 10) = Sepsis with PolymyxinB infusion; Pep 2.5 (*N *= 10) = Sepsis with peptide 19-2.5 infusion; Pep 4 (*N *= 10) = Sepsis with peptide 19-4 infusion; Pep 8 (*N *= 10) = Sepsis with peptide 19-8 infusion; Data given as mean ± SEM; *P < 0.05 versus sham. SEM, standard error of the mean.

CD14-expression in heart tissue was 3.5 ± 1.8 (mean ± SEM) fold higher in the sepsis-control compared to the sham group (*P *< 0.05). CLP resulted in a non-significant 2.0 ± 1.0 fold gene regulation in the polyB group and 1.5 ± 0.9 in the Pep 2.5-group, whereas both Pep 4 and Pep 8 showed a significant up-regulation of CD14 (4.5 ± 2.9; *P *< 0.05 and 4.9 ± 2.7; *P *< 0.05) compared to the sham group. Only the Pep 2.5-group had significantly decreased CD14 expression compared to the control (*P *= 0.003; Figure 3).

**Figure 4:**
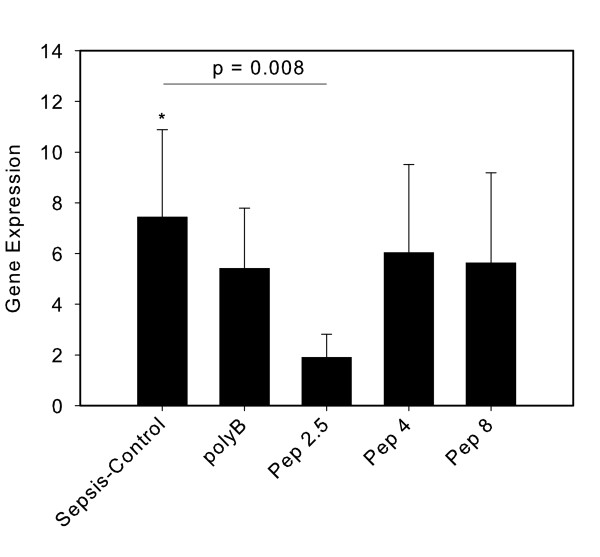
**CD14 expression in lung tissue after 24 hours of sepsis**. Sham (*N *= 10) = Sham operation with vehicle infusion; Sepsis-control (*N *= 10) = Sepsis with vehicle infusion; polyB (*N *= 10) = Sepsis with PolymyxinB infusion; Pep 2.5 (*N *= 10) = Sepsis with peptide 19-2.5 infusion; Pep 4 (*N *= 10) = Sepsis with peptide 19-4 infusion; Pep 8 (*N *= 10) = Sepsis with peptide 19-8 infusion; Data given as mean ± SEM; *P < 0.05 versus sham. SEM, standard error of the mean.

In lung tissue the pattern of CD14 expression after treatment was similar to that in the heart. The highest expression was detected in the sepsis-control (7.4 ± 3.5; *P *< 0.05) compared to the sham group. The PolyB group had a non-significant increase (5.4 ± 2.4). Pep 2.5 treatment resulted in a 1.9 ± 0.9 fold expression, thus significantly decreased compared to the sepsis-control group (*P *= 0.008). Both the Pep 4 and Pep 8 groups showed a comparable non-significant increase of CD14 (6.0 ± 3.5 and 5.6 ± 3.6, respectively) (Figure 4).

**Figure 5:**
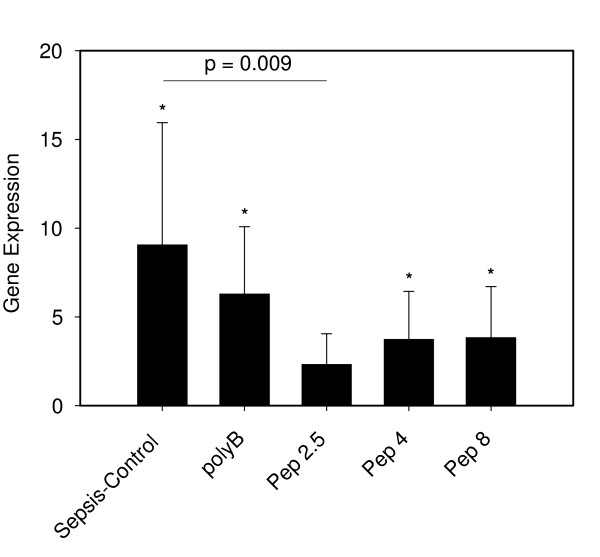
**CD14 expression in spleen tissue after 24 hours of sepsis**. Sham (*N *= 10) = Sham operation with vehicle infusion; Sepsis-control (*N *= 10) = Sepsis with vehicle infusion; polyB (*N *= 10) = Sepsis with PolymyxinB infusion; Pep 2.5 (*N *= 10) = Sepsis with peptide 19-2.5 infusion; Pep 4 (*N *= 10) = Sepsis with peptide 19-4 infusion; Pep 8 (*N *= 10) = Sepsis with peptide 19-8 infusion; Data given as mean ± SEM; *P < 0.05 versus sham. SEM, standard error of the mean.

In spleen we detected maximum CD14 expression in the sepsis-control (9.1 ± 6.7; *P *< 0.05) compared to the sham group (Figure 5). Significantly increased levels compared to the sham animals were found in the polyB, Pep 4 and Pep 8 groups (polyB: 6.3 ± 3.8; Pep 4: 3.7 ± 2.7; Pep 8: 3.8 ± 2.9; all *P *< 0.05). Pep 2.5 treatment resulted in a significantly decreased CD14 expression compared to sepsis-control (2.3 ± 1.7; *P *= 0.009).

**Figure 6:**
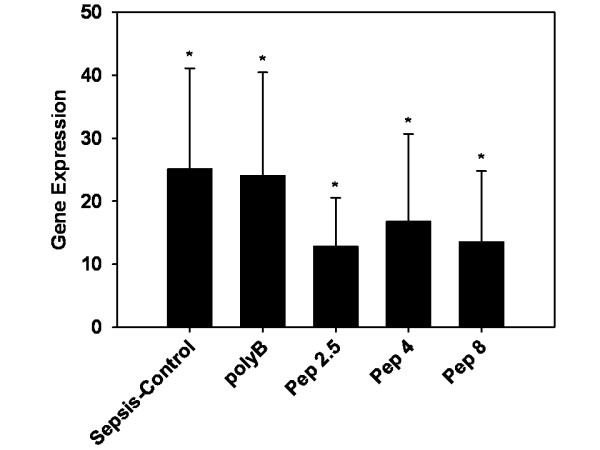
**CD14 expression in liver tissue after 24 hours of sepsis**. Sham (N 10) = Sham operation with vehicle infusion; Sepsis-control (*N *= 10) = Sepsis with vehicle infusion; polyB (*N *= 10) = Sepsis with PolymyxinB infusion; Pep 2.5 (*N *= 10) = Sepsis with peptide 19-2.5 infusion; Pep 4 (*N *= 10) = Sepsis with peptide 19-4 infusion; Pep 8 (*N *= 10) = Sepsis with peptide 19-8 infusion; Data given as mean ± SEM; *P < 0.05 versus sham. SEM, standard error of the mean.

The regulation of CD14 in liver tissue was significantly increased in all groups compared to sham treated mice (all *P *< 0.05). The sepsis-control and polyB groups showed comparable results (25.1 ± 16.0 and 24.1 ± 16.4, respectively; Figure 6). Treatment with Pep 2.5 resulted in 12.8 ± 7.7 fold expression and was not significantly different compared to control (*P *= 0.15). After Pep 4 infusion CD14 expression in liver tissue was 16.8 ± 13.9 and after Pep 8 infusion it was 13.6 ± 11.3 (Figure 6).

**Figure 7:**
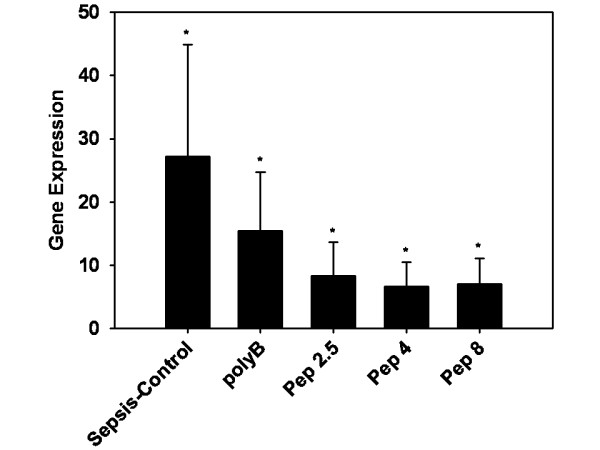
**CD14 expression in kidney tissue after 24 hours of sepsis**. Sham (*N *= 10) = Sham operation with vehicle infusion; Sepsis-control (*N *= 10) = Sepsis with vehicle infusion; polyB (*N *= 10) = Sepsis with PolymyxinB infusion; Pep 2.5 (*N *= 10) = Sepsis with peptide 19-2.5 infusion; Pep 4 (*N *= 10) = Sepsis with peptide 19-4 infusion; Pep 8 (*N *= 10) = Sepsis with peptide 19-8 infusion; Data given as mean ± SEM; *P < 0.05 versus sham. SEM, standard error of the mean.

The up-regulation of kidney derived CD14 was significantly increased in all groups (*P *< 0.05). Compared to the sham group, the sepsis-control group showed a 27.1 ± 17.8 fold increase. In the polyB group there was a significant up-regulation of 15.5 ± 9.2. All peptide treated groups revealed significantly higher CD14 expression (Pep 2.5: 8.3 ± 5.3; Pep 4: 6.6 ± 3.9; Pep 8: 7.0 ± 4.1), but showed no significant differences compared to the control (Figure 7).

## Discussion

In a model of early murine sepsis, we demonstrate that infusion of the newly synthesized Pep 2.5 is able to decrease pro-inflammatory plasma cytokine release and to decrease CD14 mRNA tissue expression compared to untreated controls.

Naturally occurring AMPs are capable of neutralizing microbial immunostimulatory cell wall constituents such as LPS. Through binding to LPS they change the aggregate structure of LPS, thereby preventing the binding to LPS-binding protein (LBP) and CD14 and subsequently to the TLR4/MD2 receptor complex [11,12]. Recombinant forms of naturally occurring AMP have been administered i.v. in sepsis without causing harm [13], but most synthetic AMPs were applied locally probably due to intrinsic toxicity [14,15]. The response to external stimulation was significantly improved in septic mice treated with SALP 19-2.5 (Table 3).

In addition, inhibition of pro-inflammatory cytokine release may provide a suitable approach to anti-infective therapy [14]. The naturally derived bactericidal/permeability increasing (BPI) protein was reported to attenuate inflammation and systemic effects caused by endotoxin release [5]. In contrast, designed AMP may exert a broader range of anti-microbial activity and may be optimized with respect to binding capacity, thus allowing smaller amounts of infused peptide.

Constitutive AMP expression levels may be enhanced by cytokines, such as IL-6 [16]. In contrast, by employing synthetic anti-inflammatory peptides, we could demonstrate a significant (85%) reduction of serum IL-6 24 hours after CLP following therapy with Pep 2.5 (Figure 2). The two other peptides (Pep 4 and Pep 8) differed in their ability to neutralize LPS and had a weaker effect on cytokine levels after challenge [4]. The different SALPs displayed the following sequence in inhibition efficiency: Pep 2.5 (very high) > Pep 4 (medium) > Pep 8 (low). These findings are in line with our results showing a non-significant decrease of IL-6 after CLP with Pep 4 (-43%) or Pep 8 (-4%) treatment (Figure 2). The different effects of the single peptides can be explained by their varying strengths of binding to bacterial cell envelope compounds. To optimize this binding, the length of the peptide, the number of basic amino acids, and the number of hydrophobic amino acids have been adapted to the physico-chemistry of LPS and LP. The peptides were designed and constructed with this aim, and Pep 2.5 was found to represent an optimum. The designed peptides act via a Coulomb interaction between peptide and LPS and a hydrophobic interaction of the amino acids at the C-terminal end of the peptides with the acyl chain moiety of LPS or LP. Both steps are necessary for this interaction [4]. Beside the LPS-binding capacity, the cytokine decrease may be partly explained by better bacterial clearance in blood and organ tissue after administration of AMP as demonstrated previously [17,18].

After incorporation in cell membranes, the peptides apparently act at the site of membrane receptors, such as CD14 [19]. Within organ tissue, CD14 is expressed by macrophages, dendritic cells, epithelial cells, endothelial cells and fibroblasts and is released into the cell supernatant or attached to the plasma membrane by a Glycosylphosphatidylinositol (GPI) anchor [20]. CD14 significantly contributes to the TLR4-mediated LPS recognition but is also involved in the stimulation by TLR2 ligands. As we used a model of polymicrobial (gram-positive and-negative) sepsis, changes in CD14 expression, increased by TLR4- and/or TLR2-mediated signaling, may reflect infection with both gram stain groups. Expression of CD14 itself is upregulated by innate immune stimulation and, thus, can be used to analyze both innate immune signaling and immune cell recruitment. Pep 2.5 therapy in CLP-induced sepsis decreased CD14 expression in lung tissue, indicating reduced innate immune stimulation and/or reduced immune cell recruitment as a consequence of Pep 2.5 mediated inhibition of inflammation (Figure 4).

The importance of CD14 for a pro-inflammatory response in heart tissue was emphasized in a mouse model, where CD14-deficient mice experienced no ventricular dysfunction and decreased cytokine mRNA expression after LPS challenge [21]. Compared to the sepsis-control group we could also demonstrate significantly decreased CD14-expression in heart tissue after Pep 2.5 therapy (Figure 3), whereas no difference was noted in liver tissue between the groups examined (Figure 6). In contrast, hepatic CD14 was previously found to be increased after LPS injection [22], and following CLP [23]. Also, repeated injections of recombinant bactericidal/permeability-increasing protein (rBPI21) in early sepsis after CLP in rats led to a significant decrease in CD14mRNA in liver, lung and kidneys [24]. Both BPI and Pep 2.5 contain a high-affinity binding domain for the lipid A component of endotoxin. Our peptides and BPI block endotoxin binding to CD14, thus inhibiting cytokine release [19,25].

Differences of mouse strains, experimental protocols and the severity of CLP may influence these results.

A strong increase of CD14 after LPS challenge and in CLP sepsis was noted in kidney tissue, predominantly in proximal tubular cells [24,26]. We could demonstrate the strongest CD14 upregulation of all investigated organs in kidney tissue. However, only non-significantly decreased expression of CD14 was noted in all treatment groups (Figure 7). Why the immunomodulatory activity of Pep 2.5 did not exert a significant effect in renal tissue is unclear. Since the anti-inflammatory effects of the tested SALPs are dose-dependent [4], one possible explanation might be the limited penetration into renal tissue. In contrast to liver and kidney, spleen tissue displayed a significant reduction of CD14 expression after Pep 2.5 treatment but not with the other tested peptides compared to controls (Figure 5). Since LPS-challenge resulted in an increase in cytokines in the spleen [27], Pep 2.5 might contribute to reduce the inflammatory reaction in spleen tissue.

Polymyxin B is considered the most potent anti-endotoxin agent and is used in experimental studies to compare endotoxin-neutralizing effects [28]. Furthermore, CD14 is not only involved in Gram-negative infection, but plays a role in lipoteichoic acid (LTA)-induced cytokine release by Gram-positive pathogens [29]. In our model we could identify gram-positive and -negative bloodstream invasion by PCR based diagnosis (Methicillin-susceptible *Staphylococcus aureus, Escherichia coli*, and *Enterococcus faecalis*, VYOO^®^, SIRS-Lab, Jena, Germany). Pep 2.5 therapy resulted in less cytokine release than polymyxin B and reduced CD14 expression in all organ tissues, suggesting activity also against Gram-positive pathogens in our model.

Yet, there are some limitations of our study. First, the use of a mouse model limits the transferability to human sepsis. Second, the 24-hour duration of our model describes only the early phase of sepsis. The results may differ in later stages of sepsis after a first improvement. Moreover, we did not provide outcome data to prove a sustained effect of continuous SALP infusion. Third, the infusion of the tested peptides started right after the sepsis stimulus, reflecting more experimental than real conditions. Furthermore, the underlying mechanisms of the effects of the tested SALP's beyond isolated gram-negative sepsis remain to be determined. An investigation on peptide activity against viruses showed that the peptides used in our study bind to heparan sulfate (HS) moieties on cells and may inhibit infection with enveloped viruses [30]. The mode of action the SALPs presented here is, therefore, not restricted to the binding to the lipid A moiety of LPS. The SALP's mode of action is innovative because it does not solely block the pro-inflammatory cascade by inactivating LPS-mediated cytokine release but possibly counters inflammation in organ tissue by interacting with HS as part of the glycocalyx. Thus, we will further investigate peptide-HS interaction in bacterial infection to clarify a general interaction.

## Conclusions

Using a widely used mouse model of polymicrobial sepsis, we analyzed the clinical efficacy of the newly developed SALP 19-2.5. Continuous Pep 2.5 infusion significantly enhanced the activity and decreased plasma levels of pro-inflammatory cytokines as well as CD14 mRNA tissue expression compared to untreated controls. Thus, SALP 19-2.5 may have the potential for further development as a tool in anti-infection treatment.

## Key messages

• Continuous parenteral administration of SALP is able to reduce sepsis-induced cytokine release.

• SALPs differ in their ability to decrease cytokine levels after sepsis challenge according to their amino acid sequence.

• CD14 mRNA tissue expression is significantly decreased by continuously infused SALP compared to untreated controls.

## Abbreviations

AMP: antimicrobial peptide; BW: body weight; CBA: cytokine bead array; CLP: cecal ligation and puncture; CVC: central venous catheter; GP: glycosylphosphatidylinositol; HR: heart rate; HS: heparin sulphate; IL: interleukin; LP: lipoprotein; LPS: lipopolysaccharide; MAP: mean arterial pressure; MCP-1: monocyte chemoattractant protein-1; NMRI: Naval Medical Research Institute; PCR: polymerase chain reaction; Pep 2.5: peptide 19-2.5; Pep 4: peptide 19-4; Pep 8: peptide 19-8; PolyB: polymyxin B; rBPI 21: recombinant bactericidal permeability increasing protein 21; SALP: synthetic anti-lipopolysaccharide peptide; TLR: Toll-like receptor.

## Competing interests

GM has received honoraria for consulting or lecturing, and restricted research grants from the following companies: BBraun, Edwards Life Sciences, Serumwerke Bernburg, Hutchinson Technology, Baxter. TPS has received travel grants from BBraun and Serumwerke Bernburg. KB has an applied patent for the structure of the synthetic antimicrobial peptide 19-2.5: Patent-No:PCT/EP2009/002565. All the other authors declare that they have no competing interests.

## Authors' contributions

TS and SD made substantial contributions to conception and design of the study, acquisition of data, analysis and interpretation of data and wrote the manuscript. MH made substantial contributions to the interpretation of data, helped to draft the manuscript and revised it critically. LM, TPS and LH have made substantial contributions to the design of the study and the acquisition, analysis and interpretation of the data. KB has made substantial contributions to the conception and design of the study and designed and provided the synthetic peptides. GM has made substantial contributions to the conception and design of the study, and to the draft of the manuscript. All authors have read and approved the final manuscript.
